# AI and Smart Devices in Cardio-Oncology: Advancements in Cardiotoxicity Prediction and Cardiovascular Monitoring

**DOI:** 10.3390/diagnostics15060787

**Published:** 2025-03-20

**Authors:** Luiza Camelia Nechita, Dana Tutunaru, Aurel Nechita, Andreea Elena Voipan, Daniel Voipan, Ancuta Elena Tupu, Carmina Liana Musat

**Affiliations:** 1 Faculty of Medicine and Pharmacy, ‘Dunarea de Jos’ University of Galati, 800008 Galati, Romania; 2 Faculty of Automation, Computers, Electrical Engineering and Electronics, ‘Dunarea de Jos’ University of Galati, 800008 Galati, Romania

**Keywords:** artificial intelligence, cardio-oncology, cardiotoxicity, machine learning, deep learning, wearable devices, implantable devices, real-time monitoring, cardiovascular risk

## Abstract

The increasing prevalence of cardiovascular complications in cancer patients due to cardiotoxic treatments has necessitated advanced monitoring and predictive solutions. Cardio-oncology is an evolving interdisciplinary field that addresses these challenges by integrating artificial intelligence (AI) and smart cardiac devices. This comprehensive review explores the integration of artificial intelligence (AI) and smart cardiac devices in cardio-oncology, highlighting their role in improving cardiovascular risk assessment and the early detection and real-time monitoring of cardiotoxicity. AI-driven techniques, including machine learning (ML) and deep learning (DL), enhance risk stratification, optimize treatment decisions, and support personalized care for oncology patients at cardiovascular risk. Wearable ECG patches, biosensors, and AI-integrated implantable devices enable continuous cardiac surveillance and predictive analytics. While these advancements offer significant potential, challenges such as data standardization, regulatory approvals, and equitable access must be addressed. Further research, clinical validation, and multidisciplinary collaboration are essential to fully integrate AI-driven solutions into cardio-oncology practices and improve patient outcomes.

## 1. Introduction

Cardio-oncology is an emerging interdisciplinary field that focuses on the prevention, diagnosis, and management of cardiovascular complications associated with cancer therapies [1,2,3]. While significant advancements in oncology have improved cancer survival rates, many of these treatments, including chemotherapy, radiation, targeted therapies, and immunotherapies, can induce cardiotoxicity [4,5,6,7,8,9], leading to heart failure, arrhythmias, hypertension, and other cardiovascular diseases [10,11,12]. The growing number of cancer survivors facing long-term cardiac complications underscores the need for innovative approaches to enhance cardiovascular risk assessment, early detection, and real-time monitoring [13,14,15].

Artificial intelligence (AI) has emerged as a transformative technology in modern medicine, offering powerful tools for data-driven decision-making [16,17]. In cardio-oncology, AI models leverage machine learning (ML), deep learning (DL), and natural language processing (NLP) to analyze vast amounts of clinical, imaging, and biomarker data [18,19]. These technologies enable the early detection of cardiotoxicity, optimize risk stratification, and provide predictive insights for personalized cardiovascular care [20]. AI-driven decision support systems are being increasingly integrated into clinical workflows, helping clinicians detect cardiovascular risks earlier and tailor interventions more effectively [21,22,23].

Alongside AI, the development of smart cardiac devices has revolutionized real-time cardiovascular monitoring [24]. Wearable technologies, such as ECG patches, smartwatches, and biosensors, allow for the continuous, remote tracking of patients’ cardiac health, reducing the need for in-hospital assessments [25]. Implantable cardiac electronic devices (ICEDs), including pacemakers, defibrillators, and cardiac resynchronization therapy (CRT) devices, are now equipped with AI-driven analytics that optimize cardiac function and predict adverse events before they become critical [26,27]. These advancements offer new possibilities for early intervention, thus improving patient outcomes and reducing the burden of cardiovascular disease in cancer patients [28,29].

Despite these innovations, challenges remain in integrating AI and smart devices into cardio-oncology. Ethical concerns such as data privacy, algorithmic bias, and disparities in access to AI-driven healthcare solutions must be addressed [30]. Additionally, clinical validation through large-scale studies and interdisciplinary collaboration between oncologists, cardiologists, data scientists, and healthcare policymakers is necessary for the widespread adoption of these technologies [31].

The objective of this review is to examine the integration of AI and smart devices in cardio-oncology, focusing on their clinical applications, challenges, and future directions. The paper is structured as follows: Section 2 discusses AI applications in cardio-oncology, Section 3 explores implantable cardiac devices, Section 4 reviews wearable smart cardiac devices, and Section 5 provides perspectives and future directions.

## 2. Methods and Materials

**Figure 1:**
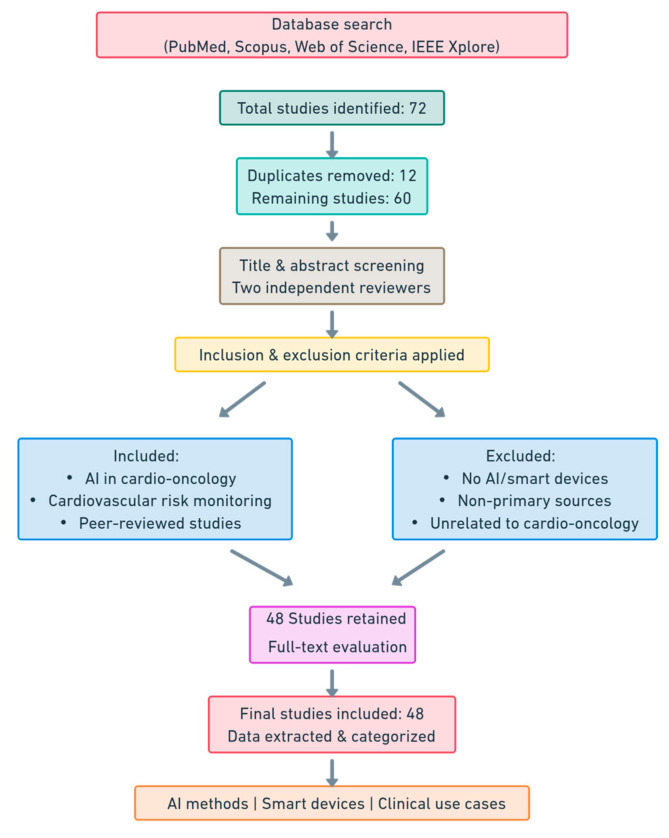
Visual roadmap of search strategy and literature selection process.

This literature review employed a comprehensive approach to identify and synthesize relevant studies on the integration of AI and smart devices in cardio-oncology. A visual roadmap is presented in Figure 1. Searches were conducted across PubMed, Scopus, Web of Science, and IEEE Xplore databases for studies published between January 2010 and December 2024. The search strategy included keywords such as “artificial intelligence”, “machine learning”, “deep learning”, “cardio-oncology”, “cardiotoxicity”, “smart devices”, “wearables”, and “implantable devices”. Boolean operators (AND, OR) were applied to refine search results and ensure a broad yet focused retrieval of the relevant literature.

The inclusion criteria targeted studies that explored AI applications in cardio-oncology, focusing on cardiovascular risk prediction, monitoring, and management. Research on smart cardiac devices, including wearable monitors and implantable devices, was also included. Only peer-reviewed journal articles, systematic reviews, clinical trials, and meta-analyses published in English were considered. Studies were excluded if they did not focus on cardio-oncology, lacked AI or smart device components, or were non-primary sources such as editorials or commentaries.

An initial search identified 72 studies, of which 12 were duplicates. After their removal, a total of 60 unique studies were retained for detailed analysis. Two independent reviewers screened titles and abstracts, followed by a full-text evaluation to determine their relevance. Any discrepancies in the study selection process were resolved through discussion with a third reviewer.

Data extraction focused on capturing key study characteristics, including research design, AI methodologies, smart device types, clinical applications, and reported outcomes. AI technologies identified in the included studies ranged from ML algorithms to DL-based medical imaging and NLP applications for electronic health records. Smart devices included wearable ECG monitors, smartwatches, and implantable cardiac devices used for cardiovascular health monitoring in oncology patients.

Since this was a comprehensive literature review rather than a systematic review, no formal risk-of-bias assessment tools such as PRISMA were applied. Instead, studies were evaluated based on relevance, study design, sample size, and applicability to cardio-oncology. The included studies were categorized based on AI methodologies, smart device applications, and clinical use cases, with a focus on early cardiotoxicity risk prediction, real-time cardiovascular monitoring, and personalized treatment strategies.

As this review relied on the publicly available literature, ethical approval and informed consent were not required. However, ethical concerns such as AI-driven healthcare disparities, patient data privacy, and algorithmic bias were critically analyzed within the context of the included studies.

After a rigorous selection and analysis process, 48 unique studies were included in this review. The findings demonstrate the transformative potential of AI and smart cardiac devices in cardio-oncology, particularly in improving cardiovascular risk prediction, enhancing real-time patient monitoring, and optimizing therapeutic interventions. Future research should focus on validating AI-driven models in clinical settings, addressing ethical concerns, and ensuring equitable access to these emerging technologies to maximize their impact on patient outcomes.

## 3. Artificial Intelligence in Cardio-Oncology

### 3.1. Introduction to AI

AI has become an essential component of modern medicine, transforming the way we diagnose, treat, and monitor patients [32]. In cardio-oncology, AI provides innovative solutions for the early detection of cardiotoxicity, risk stratification, and the optimization of therapeutic interventions [33]. The evolution of AI technologies has enabled the use of advanced ML algorithms and the processing of complex medical data, facilitating real-time data-driven clinical decision-making [22].

The integration of AI in cardio-oncology is driven by the need to manage the large volume of data generated from multiple sources such as electronic health records, medical imaging, ECG data [33], serum biomarkers, and patient clinical history. AI models can identify subtle patterns and correlations invisible to traditional methods, enabling the early detection of cardiovascular risks associated with cancer treatment.

The main uses of AI in cardio-oncology include the following:

- Cardiotoxicity risk prediction: ML models can analyze clinical and imaging data to estimate the likelihood of cardiovascular complications before, during, or after oncology treatment [34];
- Real-time patient monitoring: Smart wearables and AI systems integrated into remote monitoring allow for the early detection of relevant physiological changes [23];
- Personalization of treatment: AI helps to adjust doses and choose the most appropriate therapeutic regimens for each patient, minimizing the risk of cardiac toxicity without compromising the efficacy of oncology treatment [22];
- Automated imaging analysis: DL algorithms can analyze echocardiograms, cardiac MRIs, and other medical images to identify structural or functional changes in the myocardium [35];

The use of AI in cardio-oncology is a growing field, with numerous studies highlighting the advantages of these technologies in improving patient prognoses. As AI algorithms become more sophisticated and are integrated into clinical workflows, they will contribute significantly to optimizing the care of oncology patients at cardiovascular risk.

### 3.2. AI Technologies and Algorithms Utilized in Cardio-Oncology

AI encompasses a vast set of technologies and algorithms that can analyze complex data, detect hidden patterns, and generate accurate predictions [36]. In cardio-oncology, AI is increasingly being used to prevent, diagnose, and manage cardiovascular complications associated with cancer treatment. By leveraging AI-powered models, clinicians can enhance risk assessment, optimize monitoring, and improve therapeutic decision-making for patients undergoing oncological therapies.

Among the main AI technologies applied in cardio-oncology are ML, DL, and NLP [37]. ML algorithms enable predictive modeling by learning from clinical and imaging data, allowing for cardiotoxicity prediction and cardiovascular risk stratification. DL, a subset of ML, utilizes complex artificial neural networks (ANNs) to analyze medical images and physiological signals, making it highly effective for echocardiographic analysis and ECG abnormality detection [38]. NLP facilitates the automatic interpretation of medical documentation, enabling the efficient extraction of relevant information from electronic health records and the research literature.

**Figure 2:**
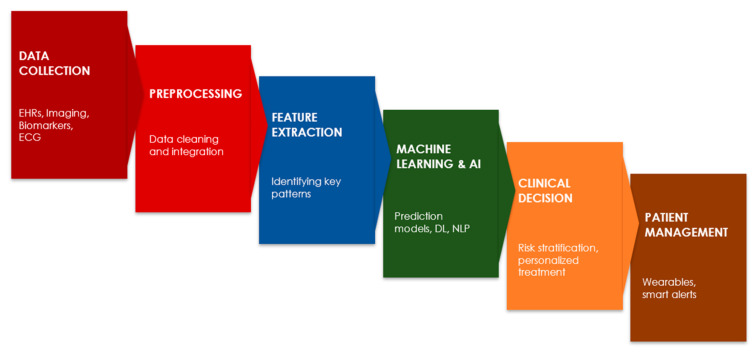
Roadmap for integrating AI technologies in cardio-oncology for risk assessment and patient monitoring.

By integrating AI-driven technologies into cardio-oncology (Figure 2), healthcare professionals can enhance the early detection of cardiovascular risks, personalize treatment plans, and ensure the continuous monitoring of patients undergoing cancer therapy. As these technologies continue to evolve, they hold the potential to revolutionize patient care by reducing the burden of cardiovascular complications associated with cancer treatments.

#### 3.2.1. Machine Learning (ML)

ML is a branch of AI that allows computer systems to learn from data and make predictions or decisions without being explicitly programmed for each possibility. Instead of following rigid instructions, ML algorithms analyze large volumes of data to identify patterns, correlations, and trends, becoming increasingly accurate as they are trained with more information [39].

A fundamental aspect of ML is its ability to extract knowledge from data and use it to make future inferences. ML algorithms are generally divided into three main categories: supervised learning, unsupervised learning, and reinforcement learning.

In supervised learning, the model is trained using a labeled dataset, which means that each input has an associated correct output [40]. The algorithm learns by comparing its own predictions with the actual results and adjusting its parameters to minimize errors. This type of ML is commonly used in classification (e.g., diagnosing a disease based on symptoms) and regression (e.g., estimating the probability of an adverse event based on a patient’s medical history). Examples of algorithms used in supervised learning include linear regression, logistic regression, decision trees, and ANNs.

In unsupervised learning, the algorithm works with unlabeled data and tries to discover hidden structures in the data [41]. Unlike supervised learning, where the model is guided by known outcomes, in unsupervised learning, the algorithm discovers patterns and relationships without a predetermined direction. This approach is commonly used for clustering patients with similar characteristics, anomaly detection (e.g., identifying patients at high risk of cardiovascular complications), and principal component analysis (PCA), a technique used to reduce the size of complex datasets.

A third category is reinforcement learning [42], where an algorithm learns by interacting with an environment and receiving a system of rewards or penalties based on the decisions made. This type of learning is inspired by the way humans make decisions and is used in complex scenarios such as optimizing personalized treatment strategies or adaptive therapy management for cancer patients.

ML plays a key role in analyzing and interpreting large volumes of medical data. In cardio-oncology, ML algorithms are used for cardiotoxicity risk prediction, the early detection of cardiovascular complications, and treatment personalization. As ML models become more advanced and are integrated into clinical systems, they have the potential to significantly improve decision-making and patient care.

#### 3.2.2. Deep Learning (DL)

DL is an advanced subcategory of ML that uses ANNs to analyze and understand complex data [43]. Unlike classical ML methods, which require manual feature engineering, DL models can automatically learn relevant representations from raw data, such as medical images, ECG signals, or clinical text. This makes DL particularly effective in analyzing large volumes of medical data, where traditional models might have difficulty identifying subtle patterns.

The NNs used in DL consist of multiple processing layers, called hidden layers, which allow the model to extract high-level features from the data. These layers are organized hierarchically so that each layer processes information and passes an intermediate result to the next layer, where it is further refined and analyzed. The greater the number of layers, the more the model is able to recognize complex structures in the data.

There are several types of NNs used in DL, each with specific applications in cardio-oncology:

- Convolutional Neural Networks (CNNs): these are mainly used for analyzing medical images such as echocardiograms, cardiac MRIs, and chest CT scans. These networks are specifically designed to detect spatially relevant features such as structural changes in the myocardium. In cardio-oncology, CNNs can identify early signs of chemotherapy-induced cardiotoxicity by automatically analyzing ultrasound or MRI images [44].
- Recurrent Neural Networks (RNNs) and Long Short-Term Memory (LSTM): these networks are used for analyzing time series, such as ECG signals or variations of serum biomarkers over time. Unlike classical NNs, RNNs can learn from data sequences, considering the temporal context. In cardio-oncology, RNN or LSTM-based models can monitor changes in patients’ cardiac parameters and predict the risk of heart failure or arrhythmias associated with oncologic treatment [45]. Automated imaging analysis: DL algorithms can analyze echocardiograms, cardiac MRIs, and other medical images to identify structural or functional changes in the myocardium;
- Autoencoding and Generative Adversarial Neural Networks (GANs): these models are used to generate synthetic data or to improve the quality of existing data. For example, autoencoders can be used to reduce noise in ECG data or medical images, improving the accuracy of automated analysis. GANs can be used to generate synthetic ultrasound images that can help train other AI models [46].

DL plays an essential role in cardio-oncology due to its ability to analyze highly complex medical data with high accuracy. For example, CNN models applied on ECG images have demonstrated the ability to detect cardiac abnormalities even before they are visible in standard clinical interpretations [47]. In addition, the integration of NNs with other types of clinical data, such as medical history and serum biomarkers, allows for the creation of more effective predictive models for cardiovascular risk stratification in oncology patients [48].

As DL technologies evolve, they will continue to transform cardio-oncology, facilitating more accurate diagnosis, more effective monitoring, and better personalization of treatments [49]. Although the clinical implementation of these models requires further validation and integration with the existing infrastructure, DL has the potential to become an indispensable tool in the care of oncology patients at cardiovascular risk.

### 3.3. Study Limitations and Methodological Challenges

Despite the promising potential of AI and smart devices in cardio-oncology, several methodological limitations must be considered. One major challenge is the lack of large-scale clinical validation. Many AI models are developed and tested on retrospective datasets, which may not accurately represent real-world clinical settings. Without prospective studies and randomized controlled trials, the clinical utility of these technologies remains uncertain.

Another key limitation is the variability in AI algorithm performance. Differences in data preprocessing, feature selection, and model architecture can lead to inconsistencies in model accuracy and reproducibility across different studies. Standardized methodologies and benchmark datasets are needed to ensure AI applications provide reliable and comparable results.

Limited accessibility to AI technologies is also a concern. The implementation of AI-driven cardio-oncology solutions requires significant investment in computational infrastructure and expertise, which may not be available in all healthcare settings. This disparity in access can widen healthcare inequities, particularly in low-resource regions.

Finally, there is a risk of over-reliance on AI-based decision-making. While AI can enhance diagnostic accuracy and streamline workflows, it should complement rather than replace clinical judgment. Physicians must remain actively involved in interpreting AI-generated insights to ensure optimal patient care.

### 3.4. Clinical Applications of AI in Cardio-Oncology

#### 3.4.1. Cardiovascular Toxicity Risk Stratification Before Oncological Treatment

Cardiac toxicity remains a major challenge in oncological practice, particularly in the context of anthracycline administration and targeted therapies such as trastuzumab [50,51]. The early identification of patients at high risk for cardiovascular complications before initiating oncological treatment is essential for optimizing clinical management and improving prognosis [52]. In this regard, AI has become an invaluable tool for risk stratification, leveraging ML and DL algorithms to achieve more accurate predictions of cancer therapy-related cardiac dysfunction (CTRCD) compared to conventional methods [53,54].

Recent studies support the effectiveness of AI-based models in cardiovascular risk estimation. In a notable example [55], predictive ML models were developed using extensive longitudinal datasets, achieving high accuracy in anticipating CTRCD by integrating clinical, laboratory, and echocardiographic variables. Similarly, another study [56] demonstrated that ML algorithms could predict cardiotoxicity in breast cancer patients treated with anthracyclines, successfully identifying high-risk individuals even before therapy initiation.

Significant advancements have also been made in AI-assisted ECG analysis. For instance, another paper [57] proposed a DL model that processes ECG images and promptly detects early signs of cardiac dysfunction, offering a non-invasive, scalable, and efficient method for patient stratification. In the same direction, a study employed AI for ECG signal analysis in predicting chemotherapy-induced cardiotoxicity [58], highlighting the potential of ECG as a screening tool for identifying vulnerable patients before starting oncological treatment.

AI-driven approaches significantly enhance clinical decision-making by integrating real-time patient data, reducing reliance on costly imaging investigations, and enabling proactive interventions aimed at mitigating cardiotoxicity risk (Table 1). Moreover, personalized therapeutic strategies tailored to the individual patient profile become more feasible, improving preventive measures and cardiac complication management. Future studies are needed to validate the robustness of these models across diverse populations, along with efforts to integrate AI technologies into routine clinical practice to optimize therapeutic outcomes in oncology.

The integration of AI in cardio-oncology has significantly improved the prediction of cardiovascular toxicity before initiating oncological treatment [63,64]. ML models now outperform traditional methods in identifying high-risk patients with greater accuracy. AI’s predictive power in cardiovascular risk assessment is significantly enhanced by integrating clinical parameters, imaging data, genetic markers, and biomarkers [65]. Combining multiple data sources provides a more comprehensive evaluation of cardiovascular health before cancer therapy [66].

Chen et al. [62] demonstrated the potential of DL in merging imaging, ECG, and biochemical data for refined risk stratification. Similarly, Madan et al. [61] highlighted AI-augmented cardiac imaging, including echocardiography, MRI, and PET scans, for the early detection of cardiotoxicity. Genetic and molecular biomarkers also contribute to risk assessment, as shown by Zheng et al. [48], who integrated genomic and proteomic data to identify patients with higher susceptibility to cardiotoxic effects.

By synthesizing diverse clinical data, AI enables personalized risk assessments, paving the way for tailored interventions. Future studies should standardize AI-integrated risk models and validate them across broader patient populations for optimal clinical use.

#### 3.4.2. Prevention and Monitoring of Cardiovascular Complications During Therapy

Cardiovascular complications during oncological therapy pose significant risks, particularly in patients undergoing anthracycline-based chemotherapy, targeted therapies, and immune checkpoint inhibitors. Traditional monitoring approaches rely on periodic imaging and biomarker assessments, which may not be sufficient for the early detection and real-time management of cardiotoxicity [67]. AI has emerged as a transformative tool in cardio-oncology, offering automated risk detection, continuous monitoring, and personalized intervention strategies to enhance patient outcomes.

Several AI-based models have demonstrated superior performance in identifying the early signs of cardiotoxicity, particularly through advanced ECG analysis and DL models. Jacobs et al. [18] developed an AI-enhanced ECG tool to detect left ventricular dysfunction post-anthracycline therapy, achieving high accuracy in identifying abnormal ejection fractions (EFs), while Yagi et al. [68] leveraged AI-enabled ECG-based predictions to assess chemotherapy-induced cardiotoxicity before clinical symptoms appear. Beyond these, AI-driven clinical decision support systems are reshaping how cardiotoxicity is managed in real time. By integrating patient similarity algorithms and ML-based decision models, AI facilitates personalized treatment modifications and exercise-based prevention strategies. Brown et al. [69] introduced an AI-powered patient similarity model to optimize shared decision-making, while Gao et al. [70] designed an intelligent system to personalize exercise prescriptions for cancer patients at risk of cardiovascular disease.

As summarized in Table 2, these AI-driven solutions enhance both the early detection and management of cardiovascular risks, providing real-time insights that allow clinicians to proactively mitigate complications during therapy.

AI is significantly improving the early detection and monitoring of chemotherapy-induced cardiotoxicity, helping to prevent irreversible cardiac damage. AI-powered ECG analysis has demonstrated high accuracy in detecting left ventricular dysfunction (LVEF reduction) before structural abnormalities become apparent. Jacobs et al. [18] achieved an AUC of 0.93 for EF < 50% using DL algorithms, outperforming standard ECG interpretation. Similarly, Halasz et al. [71] used CNNs for the automated detection of cardiac dysfunction at an early stage.

Beyond ECG, AI-enhanced imaging techniques, such as AI-assisted echocardiography, allow non-specialist oncology staff to evaluate cardiac function with high sensitivity and specificity. These tools, when integrated into clinical workflows, could revolutionize cardiotoxicity screening and enable timely interventions.

AI also plays a crucial role in real-time clinical decision support by leveraging continuous patient data. AI-driven models improve risk prediction, treatment customization, and intervention planning. Brown et al. [69] developed a patient similarity model to personalize cardiovascular prevention strategies, while Gao et al. [70] introduced an AI-based system for exercise recommendations to mitigate cardiovascular risk.

By incorporating AI into oncology practice, clinicians can identify cardiovascular risks earlier, personalize treatment, and improve long-term outcomes for cancer patients. AI-driven multimodal integration will further refine patient monitoring, ultimately enhancing preventive strategies and reducing cardiovascular disease burden in cancer survivors.

#### 3.4.3. Diagnosis and Management of Acute and Subacute Cardiovascular Toxicity

Acute and subacute cardiovascular toxicity remains a significant concern in oncology, particularly among patients receiving anthracyclines, trastuzumab, and immune checkpoint inhibitors [74]. These cardiotoxic effects can manifest as arrhythmias, left ventricular dysfunction, myocarditis, and acute heart failure, often requiring early diagnosis and immediate intervention to prevent long-term cardiac damage [75]. Traditional diagnostic methods, such as echocardiography and cardiac biomarkers, may not always detect subtle cardiac dysfunction in its early stages.

AI is increasingly being integrated into cardio-oncology workflows to enhance the detection, differentiation, and management of therapy-induced cardiovascular complications. AI-driven models automate risk stratification, analyze multimodal data sources, and improve decision-making processes, leading to earlier intervention and better clinical outcomes.

Recent studies have highlighted the use of AI-enhanced ECG and echocardiography for early cardiotoxicity screening. Oikonomou et al. [57] demonstrated that AI-driven ECG imaging could accurately identify cardiac dysfunction, while Papadopoulou et al. [73] developed an AI-assisted echocardiographic tool to enhance oncologist-led cardiac function assessment. Additionally, Zhou Y et al. [55] explored DL algorithms applied to longitudinal EHRs to predict heart disease risk in breast cancer patients, supporting data-driven clinical decision-making.

As summarized in Table 3, AI is proving to be an effective tool in both automated differential diagnosis and the optimization of cardiotoxicity management. These technologies have the potential to reduce the burden of cardiac complications, improve early risk assessment, and enhance personalized treatment strategies for cancer patients at risk of cardiovascular disease.

Beyond early detection, AI enhances treatment personalization by integrating longitudinal patient data, biomarkers, and imaging results to guide clinical decisions. Zhou S et al. [76] showed that DL applied to EHRs can predict cardiovascular disease risk in cancer patients with high accuracy, allowing for proactive adjustments in treatment plans. AI-powered telemedicine solutions, such as those explored by Kappel et al. [29], improve access to cardiotoxicity monitoring and treatment modifications, particularly for underserved populations. Additionally, AI-driven patient similarity models and real-time drug safety monitoring enable precision medicine approaches, optimizing cardioprotective interventions and minimizing toxicity risks.

By integrating AI into routine oncology practice, clinicians can improve the early detection and management of cardiovascular complications, personalize treatment strategies, and enhance long-term outcomes for cancer patients. Future research should focus on refining these AI models, expanding telehealth capabilities, and integrating multimodal data to maximize their clinical impact.

#### 3.4.4. AI in Long-Term Cardiovascular Risk Identification and Chronic Complication Management in Cancer Survivors

Cancer survivors remain at an elevated risk for long-term cardiovascular complications, which can arise years or even decades after completing oncological treatment. The adverse cardiac effects of chemotherapy, radiation therapy, and targeted therapies such as anthracyclines and immune checkpoint inhibitors may progress into chronic cardiovascular disease, including heart failure, myocardial infarction, and arrhythmias [77,78]. The need for the early identification and long-term monitoring of these late-onset cardiac risks has become a priority in cardio-oncology care.

AI offers innovative solutions for both the risk stratification and long-term management of cardiovascular complications in cancer survivors [79]. AI-driven models leverage EHRs, imaging, biomarker data, and wearable technology to track disease progression, identify high-risk patients, and personalize follow-up strategies. These tools have the potential to improve patient outcomes by facilitating earlier interventions, remote monitoring, and precision medicine approaches.

Studies have demonstrated the effectiveness of AI-enhanced ECG and multimodal imaging in detecting subclinical cardiac dysfunction. Jacobs et al. [18] highlighted the ability of AI-based ECG screening to identify patients with reduced ejection fraction following anthracycline therapy. Furthermore, AI has been integrated into telemedicine and digital health platforms to expand access to specialized cardio-oncology care in underserved populations [77].

Table 4 summarizes the key studies evaluating AI-based strategies for long-term cardiovascular risk identification and chronic complication management.

AI-driven models are improving the prediction and management of long-term cardiovascular risk in cancer survivors. ML techniques, including DL, applied to longitudinal EHR data, imaging, and biomarkers, have significantly enhanced risk assessment accuracy. Jacobs et al. [18] demonstrated that AI-ECG screening effectively detects abnormal LVEF in patients previously treated with anthracyclines, allowing for earlier cardiac monitoring. AI-enhanced ECG analysis, as reviewed by Nechita et al., reviews a cost-effective, non-invasive solution for long-term cardiac surveillance [33]. However, the continuous validation of these models is needed to ensure fairness across diverse populations, as highlighted by Echefu et al. [32].

Beyond risk prediction, AI is transforming long-term cardiovascular management in cancer survivors through telemedicine, wearable monitoring, and predictive analytics. AI-assisted remote monitoring improves access to specialized cardio-oncology care, particularly in underserved areas, as demonstrated by Kappel et al. [29]. Clinicians can track patient progress in real time using AI-powered ECG interpretation and digital biomarkers. AI-enhanced decision support tools also optimize exercise prescriptions, medication adjustments, and follow-up strategies, as shown by Brown et al. [69]. Personalized rehabilitation programs, such as those developed by Gao et al. [70], further reduce cardiovascular risk by tailoring interventions to individual patient needs.

Integrating AI into chronic cardiovascular disease management enables a proactive, data-driven approach that improves patient outcomes, minimizes complications, and ensures better long-term cardiovascular health for cancer survivors.

## 4. Implantable Cardiac Electronic Devices Used in Cardio-Oncology

### 4.1. Introduction to Implantable Devices

ICEDs play a crucial role in managing cardiovascular complications in cancer patients, particularly those undergoing cardiotoxic cancer therapies [81]. These devices support heart function, prevent arrhythmic complications, and improve survival rates in oncology patients with cardiac dysfunction. The primary types of ICEDs include cardiac pacemakers, ICDs [82], pulmonary artery pressure monitoring systems, and cardiac CRT devices.

Each device serves a distinct function tailored to specific cardiovascular conditions exacerbated by cancer treatments. Advances in technology and the integration of AI have enhanced their effectiveness in early detection, personalized therapy adjustments, and real-time monitoring.

### 4.2. Types of Implantable Devices in Cardio-Oncology

ICEDs play a critical role in managing cardiovascular complications in cancer patients, particularly those undergoing cardiotoxic treatments such as chemotherapy and radiation therapy. These devices help maintain cardiac function, monitor real-time changes in heart activity, and provide life-saving interventions for arrhythmias and heart failure [83]. The most used implantable devices in cardio-oncology include cardiac pacemakers, ICDs, CRT devices, and pulmonary artery pressure monitoring systems.

#### 4.2.1. Cardiac Pacemakers

Pacemakers are used to manage bradyarrhythmia that may arise from cancer therapies, particularly chemotherapeutic agents like anthracyclines and immune checkpoint inhibitors, which can cause sinus node dysfunction and atrioventricular block [84]. These devices regulate the heart rate by delivering small electrical impulses to stimulate myocardial contraction when the intrinsic cardiac rhythm is too slow.

A study by Riesenhuber et al. [85] examined the outcomes of pacemaker implantation in cancer patients and found that individuals who required a pacemaker after a cancer diagnosis had significantly lower 10-year survival rates compared to those who had a pacemaker implanted prior to their diagnosis. This underscores the importance of early cardiac screening and intervention in high-risk oncology patients.

#### 4.2.2. Clinical Impact of AI on ICDs

ICDs are critical for preventing sudden cardiac death in cancer patients at high risk for ventricular tachycardia or ventricular fibrillation. These devices continuously monitor heart rhythms and deliver electrical shocks when life-threatening arrhythmias are detected [86]. Patients receiving radiation therapy to the chest, particularly for lung and breast cancers, may develop myocardial fibrosis and electrical disturbances that predispose them to fatal arrhythmias.

Studies have highlighted concerns regarding ICD function in cancer patients undergoing radiation therapy, as high-energy radiation can interfere with device programming and battery life. The close monitoring and strategic placement of ICDs in patients requiring thoracic irradiation are necessary to minimize complications [87].

#### 4.2.3. Clinical Impact of AI on CRTs

CRT devices are designed to improve heart function in patients with chemotherapy-induced cardiomyopathy or heart failure. By coordinating the contraction of the left and right ventricles, these devices enhance cardiac efficiency, particularly in patients with reduced ejection fraction and left bundle branch block [88].

Cancer survivors treated with cardiotoxic agents such as doxorubicin and trastuzumab are at risk of progressive heart failure, where CRT implantation may improve functional capacity and reduce hospitalizations. Lee SY et al. [89] analyzed the role of CRT in cardio-oncology patients, highlighting its benefits in reversing left ventricular dysfunction associated with cancer treatments.

#### 4.2.4. Clinical Impact of AI on Pulmonary Artery Pressure Monitoring Devices

Pulmonary artery (PA) pressure monitoring devices, such as CardioMEMS [90], provide real-time hemodynamic assessment in patients with cancer-related heart failure. These sensors are implanted in the pulmonary artery and wirelessly transmit pressure readings to clinicians, enabling the proactive management of fluid status and heart failure exacerbations.

Cancer therapies that induce hypertension and left ventricular dysfunction—such as VEGF inhibitors and certain immunotherapies—can lead to increased pulmonary pressure [91]. Continuous PA pressure monitoring allows for the early detection of decompensation, reducing the likelihood of heart failure-related hospitalizations.

### 4.3. Integration of AI with Implantable Devices

#### 4.3.1. Advancements in AI-Integrated Implantable Devices

One of the primary advantages of AI-enhanced implantable devices is their ability to continuously collect and process large volumes of real-time patient data. AI-driven pacemakers and ICDs can adjust their function dynamically based on patient-specific physiological changes, ensuring optimal cardiac support. For example, ML models analyze ECG and hemodynamic data from these devices to predict cardiovascular deterioration before it manifests clinically.

AI-enhanced CRT devices are now capable of optimizing ventricular pacing strategies based on individualized cardiac function metrics. This technology is particularly useful for cancer patients with chemotherapy-induced cardiomyopathy, as AI algorithms can detect early changes in cardiac conduction and guide timely adjustments in therapy to improve patient outcomes.

Additionally, wearable biosensors linked to implantable devices provide another layer of monitoring by tracking heart rate variability, oxygen saturation, and other vital signs [16,17]. These sensors transmit data to cloud-based AI platforms, allowing for the continuous assessment of a patient’s cardiovascular health. If abnormalities are detected, automated alerts are sent to healthcare providers, enabling prompt intervention.

#### 4.3.2. Clinical Impact of AI in ICEDs

The implementation of AI in ICEDs is significantly enhancing the early detection and prevention of severe cardiovascular events in oncology patients. AI-assisted monitoring reduces diagnostic delays and enhances treatment precision, mitigating the risk of sudden cardiac events associated with chemotherapy-induced cardiotoxicity. These developments allow for personalized treatment, with AI algorithms identifying patient-specific risk factors and guiding interventions accordingly.

One of the key challenges in AI-integrated ICEDs is ensuring the reliability and interpretability of AI-generated alerts. False alarms can lead to unnecessary interventions, while missed detections may delay critical treatment. Ongoing research is focused on improving the specificity and sensitivity of AI-based monitoring systems to enhance their clinical applicability (Table 5).

The future of AI-integrated implantable devices lies in expanding their predictive capabilities, refining automated interventions, and ensuring seamless interoperability with other healthcare systems. The standardization of AI algorithms and validation across diverse populations will be essential for maximizing their clinical benefits.

## 5. Wearable Smart Cardiac Devices Used in Cardio-Oncology

Wearable smart cardiac devices have emerged as essential tools in cardio-oncology, facilitating real-time monitoring and the early detection of cardiovascular complications associated with cancer treatments. These devices offer a non-invasive, continuous means of assessing cardiac function, detecting arrhythmias, and optimizing patient management strategies [24]. The integration of AI and ML further enhances their predictive capabilities, allowing for early intervention and personalized care.

### 5.1. Introduction to Wearable Devices

Wearable devices represent a rapidly evolving segment of embedded systems, integrating advanced sensing, computing, and communication technologies into compact, body-worn form factors. These devices are designed to collect, process, and transmit physiological, biomechanical, and environmental data in real time, enabling a wide range of applications in healthcare, fitness monitoring, human–computer interactions, and industrial settings.

At their core, wearable devices leverage miniaturized microcontrollers (MCUs) or system-on-chip (SoC) architectures, often featuring low-power processing capabilities to support continuous operation with minimal energy consumption [94]. They are typically equipped with a variety of integrated sensors, such as the following:

- Inertial Measurement Units (IMUs) for motion tracking and activity recognition [95];
- Photoplethysmography (PPG) sensors for heart rate and oxygen saturation monitoring [94];
- Electromyography (EMG) and Electroencephalography (EEG) sensors for muscle and neural activity analysis [96];
- Galvanic Skin Response (GSR) sensors for stress and emotional state assessment [97].

Communication modules, including Bluetooth Low Energy (BLE), Wi-Fi, NFC, and LPWAN technologies, facilitate seamless connectivity with edge devices, cloud platforms, and mobile applications [98]. Data processing in wearables can be on-device (edge AI) for real-time decision-making or offloaded to cloud infrastructures for more complex analytics and long-term storage.

Power efficiency remains a critical challenge in wearable device design, driving advancements in energy-harvesting techniques, ultra-low-power processors, and optimized wireless communication protocols. Recent innovations in flexible electronics, bio-compatible materials, and AI-driven signal processing are further expanding the capabilities of wearables, enabling applications such as continuous health monitoring, predictive diagnostics, and real-time feedback for enhanced user experience.

With the integration of ML algorithms, wearables are transitioning from passive monitoring tools to intelligent systems capable of adaptive, context-aware functionalities, significantly enhancing their role in personalized healthcare, rehabilitation, and performance optimization.

### 5.2. Advances in Wearable Cardiac Monitoring

The landscape of wearable cardiac technology has expanded rapidly in recent years, driven by advancements in sensor technology, miniaturization, and AI integration. Devices such as smartwatches, fitness trackers, and portable ECG monitors have transitioned from consumer-grade wellness tools to clinically validated medical devices. These technologies enable healthcare professionals to track HRV, detect AF, monitor blood pressure, and assess overall cardiovascular health in cancer patients undergoing therapy.

One of the key challenges in cardio-oncology is chemotherapy-induced cardiotoxicity. Anthracyclines, immune checkpoint inhibitors, and targeted therapies have been associated with an increased risk of left ventricular dysfunction and arrhythmias. Wearable devices, such as ECG patches (e.g., ZioPatch) and photoplethysmography (PPG)-based wristbands [99], allow for the real-time tracking of cardiac parameters, alerting clinicians to early signs of cardiotoxicity.

### 5.3. Clinical Applications in Cardio-Oncology

Wearable smart cardiac devices are transforming the management of cardiovascular complications in oncology patients by providing continuous, real-time monitoring of critical physiological parameters. These innovations (Table 6) support early detection, risk stratification, and intervention, reducing the burden of cardiovascular disease among cancer patients.

One of the most significant applications of wearable technology in cardio-oncology is the detection and management of arrhythmias. Cancer patients undergoing chemotherapy and radiation therapy face an increased risk of developing arrhythmias such as AF and ventricular tachycardia. Traditional monitoring methods often fail to detect transient or silent arrhythmias, which can later manifest as severe complications. Wearable ECG devices have proven effective in capturing these abnormalities, providing a non-invasive, long-term solution for at-risk patients. Devices such as KardiaMobile by AliveCor [101] and the ECG functionalities of the Apple Watch have been validated for AF detection, offering patients a user-friendly and clinically reliable means of monitoring their heart rhythm. A study conducted by Faro et al. [101] revealed that although most cancer survivors owned smartphones, only a small percentage used wearable ECG monitors. However, there was a strong patient interest in utilizing these devices for arrhythmia detection, particularly for AF. Continuous, real-time heart rhythm monitoring is particularly beneficial for immunocompromised oncology patients, reducing the need for frequent hospital visits while ensuring early intervention when necessary.

Hypertension is another cardiovascular complication frequently associated with cancer therapies, especially those involving VEGF inhibitors. These treatments can induce or exacerbate hypertension, increasing the risk of cardiovascular events such as stroke and heart failure. Wearable blood pressure monitors like Omron HeartGuide [99] provide patients with an accessible tool to track their blood pressure at home. AI-powered analytics integrated into these devices enable early detection of hypertensive trends, allowing for timely clinical interventions before a crisis occurs. This technology enhances patient autonomy while ensuring that clinicians receive real-time data to guide treatment modifications.

Beyond arrhythmia and hypertension management, wearable devices also play a crucial role in cardiotoxicity surveillance. Many cancer treatments, including anthracyclines and HER2-targeted therapies such as trastuzumab, have well-documented cardiotoxic effects, potentially leading to progressive heart failure if left undetected [23]. Monitoring early indicators of cardiotoxicity, such as HRV and autonomic dysfunction, is essential for preventing irreversible cardiac damage. Wearable ECG patches like Zio XT enable extended monitoring periods, capturing transient ischemic changes, QT prolongation, and other subclinical markers of cardiotoxicity [24]. Research indicates that AI-enhanced analysis of wearable ECG data can refine risk stratification, enabling early interventions and reducing the likelihood of treatment-induced heart failure.

Remote patient monitoring and telemedicine have gained widespread adoption, particularly in the wake of the COVID-19 pandemic [33]. Wearable devices provide continuous cardiac surveillance, transmitting real-time physiological data to healthcare providers. This technology reduces the necessity for frequent in-person visits while maintaining close clinical oversight. AI-powered platforms capable of aggregating and analyzing wearable data are being developed to enhance risk stratification and automate clinical decision-making. The ability to integrate wearable devices into telemedicine frameworks has significantly improved patient outcomes, particularly for those undergoing cancer treatment who require close cardiovascular monitoring but face logistical barriers to in-person consultations.

The integration of AI and ML has revolutionized the capabilities of wearable devices in cardio-oncology. AI-driven analytics allow for the detection of early cardiac deterioration patterns that might not be apparent through conventional diagnostic methods. Automated arrhythmia classification is one of the key functionalities enabled by DL models, distinguishing between benign palpitations and life-threatening arrhythmias with high sensitivity and specificity. AI-powered early warning systems analyze trends in heart rate, HRV, and blood pressure, flagging concerning deviations before they escalate into critical cardiovascular events. Furthermore, ML algorithms are increasingly being utilized to personalize treatment recommendations, tailoring interventions based on individual patient responses to therapy.

A study by Hughes et al. [99] underscored the potential of AI-enhanced wearable cardiac monitoring, demonstrating how ML models improve AF detection accuracy, optimize cardiovascular risk assessment, and enhance patient management strategies. However, despite these promising advancements, several barriers remain to widespread adoption. Regulatory challenges, data privacy concerns, and the need for large-scale clinical validation continue to hinder the seamless integration of AI-powered wearables into routine oncology care. Overcoming these hurdles will be crucial in harnessing the full potential of smart wearable devices to improve cardiovascular outcomes for cancer patients.

Incorporating wearable devices into cardio-oncology practice represents a paradigm shift in cardiovascular risk management, offering a proactive approach to patient monitoring. By leveraging AI-enhanced continuous monitoring solutions, clinicians can detect complications earlier, intervene more effectively, and ultimately improve the quality of life and survival rates for patients undergoing cancer treatment.

## 6. Perspectives, Future Directions, and Conclusions

### 6.1. Emerging Trends and Technological Developments

The future of cardio-oncology is being reshaped by rapid advancements in AI and smart cardiac devices. AI-powered predictive analytics, DL algorithms, and NLP are being increasingly integrated into clinical practice, enhancing the early detection and management of cardiovascular complications in cancer patients. The continued evolution of ML models enables more precise cardiotoxicity risk assessments, while AI-driven imaging analysis is improving the interpretation of echocardiograms, ECGs, and cardiac MRIs.

One of the most promising developments is the use of wearable and implantable smart devices for continuous cardiac monitoring. Smartwatches and biosensors equipped with AI-driven analytics now allow real-time tracking of heart rate variability, arrhythmias, and other cardiovascular parameters. ICEDs, including pacemakers and defibrillators, are evolving to integrate AI-driven decision support, enabling early intervention for high-risk patients. These innovations facilitate remote patient monitoring, reducing hospital visits and improving long-term cardiovascular health outcomes.

Another emerging trend is the increasing role of AI in precision medicine. By leveraging genomic, proteomic, and metabolomic data, AI can identify patients at higher risk for chemotherapy-induced cardiotoxicity, allowing for personalized treatment regimens. AI-driven decision support systems are also helping oncologists and cardiologists select optimal therapeutic strategies that balance cancer efficacy with cardiovascular safety [103]. The integration of multi-modal AI models, combining clinical history, imaging, biomarkers, and real-time physiological data, will further enhance risk prediction and treatment personalization.

In the coming years, AI integration in cardio-oncology is expected to evolve through several key pathways. Firstly, AI models will shift towards federated learning, ensuring data privacy while training on diverse, multi-institutional datasets. Secondly, smart cardiac devices will incorporate real-time feedback mechanisms, allowing for adaptive treatment plans based on continuous patient monitoring. Finally, regulatory bodies will need to establish standardized guidelines to facilitate the safe implementation of AI in clinical practice, bridging the gap between technological innovation and real-world applications. Another important aspect is the development of integrated digital ecosystems that are capable of connecting implantable and wearable devices with national or international health information systems. These ecosystems could enable the secure and rapid exchange of data between clinicians and patients, thus facilitating prompt interventions and seamless and personalized care, especially in contexts of health crises or limited direct access to health services. In this way, the widespread and standardized use of data generated by smart devices could substantially improve the quality and accessibility of cardio-oncology services globally.

In addition, the expansion of AI applications in cardio-oncology can generate important economic benefits by optimizing the use of medical resources and reducing the costs of frequent hospitalizations and emergency interventions. Another key issue is the development of explainable AI algorithms that increase clinicians’ confidence in the recommendations generated by automated systems and facilitate their integration into the daily clinical decision-making workflow.

### 6.2. Research and Multidisciplinary Collaboration

The successful implementation of AI and smart devices in cardio-oncology requires close collaboration between researchers, clinicians, engineers, and data scientists. Multidisciplinary partnerships are essential for developing robust AI models that can integrate seamlessly into existing clinical workflows. Large-scale prospective studies and real-world evidence are needed to validate AI-powered risk prediction tools and confirm their clinical utility.

One of the key priorities for future research is the standardization of data collection and interoperability across healthcare institutions. AI models rely on high-quality datasets, yet variability in clinical data acquisition, imaging protocols, and HER formats can hinder the accuracy of AI-driven predictions. Establishing universal guidelines for data sharing and model validation will enhance the reproducibility of AI applications and facilitate their broader adoption in cardio-oncology.

Ethical considerations must also be addressed through collaborative efforts. AI algorithms may inadvertently introduce biases if trained on non-representative datasets, leading to disparities in cardiovascular care among different populations. Researchers and policymakers should work together to ensure that AI-driven models are equitable, unbiased, and accessible to all patients, regardless of their socioeconomic background. Additionally, continuous education and training for healthcare professionals will be vital in ensuring that clinicians can effectively interpret AI-generated insights and incorporate them into patient management strategies.

Beyond academia, collaborations with industry partners will play a critical role in advancing AI-driven smart devices for cardio-oncology applications. Wearable and implantable device manufacturers are actively integrating AI-powered analytics into their products, but regulatory and clinical validation hurdles remain. Joint efforts between regulatory agencies, medical professionals, and technology developers will be necessary to accelerate the safe deployment of AI-powered healthcare innovations.

Moreover, future research should aim at the prospective clinical validation of AI algorithms and smart devices in real-world clinical practice scenarios in randomized, multicenter studies. There is also a need to develop clear protocols and specific international guidelines for the application of AI technologies in cardio-oncology. Such guidelines should include not only technical and clinical recommendations but also clear ethical and legal issues to ensure consistent and responsible implementation. In parallel, future research should further explore how AI and smart devices can be integrated with emerging technologies, such as blockchain for securing and standardizing medical data, and augmented reality (AR) for real-time decision support in operating rooms or interdisciplinary cardio-oncology consultations. In this regard, interdisciplinary investigations that bring together specialists in medicine, technology, bioethics, and public policy will be essential to maximize the benefits of these technological innovations in medical practice.

### 6.3. Conclusions

AI and smart cardiac devices are transforming the field of cardio-oncology, offering novel solutions for early cardiotoxicity risk prediction, real-time patient monitoring, and personalized treatment strategies. The integration of ML, DL, and NLP into clinical practice is improving the accuracy of cardiovascular risk assessment, while wearable and implantable smart devices are enabling continuous cardiac surveillance. These advancements have the potential to enhance patient outcomes by minimizing cardiovascular complications associated with cancer therapies.

Despite these promising developments, several challenges must be addressed to fully integrate AI-driven solutions into cardio-oncology. The need for high-quality, diverse datasets, model transparency, and regulatory oversight remains critical. Ethical considerations related to algorithmic bias and healthcare disparities must also be carefully managed to ensure equitable access to AI-driven care. Furthermore, ongoing research and multidisciplinary collaborations will be essential in refining AI models, standardizing clinical applications, and validating real-world effectiveness.

Looking ahead, the future of cardio-oncology will likely involve the seamless integration of AI with digital health technologies, including telemedicine, remote monitoring, and real-time decision support. As AI-powered tools continue to evolve, they will not only enhance cardiovascular care for cancer patients but will also redefine the broader landscape of precision medicine. Specifically, we anticipate that in the coming years, AI models will become a standard component of cardio-oncology instrumentation, facilitating individualized predictive assessments for each oncology patient. In addition to the early identification of vulnerable patients, these technologies will enable dynamic therapeutic adjustments that are continuously tailored to the patient’s individual cardiovascular profile. At the same time, the widespread deployment of these intelligent systems could significantly reduce the rates of severe cardiovascular complications associated with oncologic therapies, thus directly contributing to improving the long-term survival and quality of life of cancer patients. By fostering collaboration between clinicians, researchers, and technology innovators, AI and smart devices can be harnessed to optimize cardiovascular outcomes and improve the overall quality of life for oncology patients.

## Figures and Tables

**Table 1:** Scientific articles analyzing the role of AI in cardiovascular toxicity risk stratification before oncologic treatment.

Category	Year of Study	Author	ML Method	Data Source	Application	Performance Metrics
Predictive algorithms for cardiovascular risk estimation	2023	Han S et al. [59]	XGBoost, AdaBoost, Decision Tree	Nationwide Readmissions Database (358,629 hospitalized cancer patients)	Predicting unplanned readmission due to cardiovascular disease in hospitalized cancer patients	XGBoost had best predictive performance for unplanned cardiovascular readmissions
	2023	Al-Droubi SS et al. [60]	Random Forest (RF), ANN	20,023 oncology patient records from Vanderbilt University Medical Center	AI modeling for cardiovascular disease risk assessment in oncology patients	Accuracy: >90% for ANN, superior to RF
	2022	Chang WT et al. [56]	RF, Logistic Regression, LightGBM, KNN, MLP	Prospective study on breast cancer patients receiving anthracycline therapy (2014–2018)	Prediction of cardiotoxicity in breast cancer patients using ML models trained on clinical and imaging data	Best performing model: MLP (AUROC = 0.66, Sensitivity: 0.86, Specificity: 0.53)
	2020	Zhou Y et al. [55]	RF, SVM, Gradient Boosting	Electronic medical records of 4309 cancer patients (Cleveland Clinic, 1997–2018)	Risk assessment of cancer therapy-related cardiac dysfunction using clinical and echocardiographic data	AUROC: 0.821 (CAD), 0.787 (AF), 0.882 (HF), 0.807 (MI), 0.802 (de novo CTRCD)
Integration of clinical, imaging, genetic, and biomarker data	2025	Oikonomou EK et al. [57]	DL (CNN applied to ECG images)	Retrospective study of 1550 patients undergoing anthracycline or trastuzumab treatment (2013–2023, Yale New Haven Health System)	AI-enhanced ECG-based risk stratification for early detection of cardiac dysfunction post-chemotherapy	Hazard ratio for high-risk patients: 3.35 for CTRCD, 13.52 for LVEF <40%
	2024	Yagi R et al. [58]	AI-CTRCD Model (Transfer Learning on AI-EF Model, ECG-based)	Multicenter study on 1011 anthracycline-treated patients (Brigham & Women’s Hospital, Massachusetts General Hospital, Keio University Hospital)	AI-powered ECG analysis for predicting chemotherapy-induced cardiotoxicity before treatment initiation	Time-dependent AUC for 2-year prediction: 0.78 with AI-CTRCD vs. 0.74 without (*p* = 0.005)
	2023	Zheng Y et al. [48]	RF, ANN, Convolutional Neural Networks	Large-scale cardio-oncology patient registry integrating clinical and imaging data	ML-driven insights into cardio-oncology risk factors and treatment outcomes	RF and ANN demonstrated strong performance in patient risk stratification
	2022	Madan N et al. [61]	AI-Augmented Cardiac Imaging (Echocardiography, MRI, PET)	Advanced cardiac imaging databases, including echocardiography, MRI, and PET	AI-integrated imaging for early detection and prevention of cardiotoxicity in cancer patients	AI-augmented imaging models improved early detection of cardiac dysfunction
	2022	Chen H et al. [62]	DL (Multimodal Imaging)	Cardiovascular imaging and biomarker datasets (Stanford, Cedars Sinai, City of Hope)	AI-driven analysis of high-dimensional cardiovascular data for risk stratification	AI-enhanced imaging analysis improved diagnostic accuracy significantly

**Table 2:** Scientific articles analyzing the role of AI in the prevention and monitoring of cardiovascular complications during oncologic therapy.

Category	Year of Study	Author	ML Method	Data Source	Application	Performance Metrics
Early detection of cardiotoxicity	2024	Jacobs JEJ et al. [18]	DL on ECG	Single-center cohort of breast cancer patients receiving anthracyclines (N = 989)	AI ECG tool to detect abnormal left ventricular ejection fraction post-anthracycline therapy	AUC = 0.93 for EF < 50%, AUC = 0.94 for EF ≤ 35%
	2024	Halasz G et al. [71]	Convolutional Neural Networks (CNN) applied to ECG	Single-center study on AI-based ECG detection of abnormal LVEF post-anthracycline therapy	Screening for newly abnormal LVEF using AI-assisted ECG analysis	DL-based AI-ECG achieved high sensitivity and specificity in detecting LVEF abnormalities
	2022	Martinez DS et al. [16]	AI-enhanced ECG risk stratification	Review of AI opportunities in cardio-oncology with focus on ECG-based monitoring	Opportunities in cardio-oncology using AI-enhanced ECG assessment	N/A (Review article summarizing AI in ECG for cardio-oncology)
Real-time data analysis for clinical decision support	2023	Brown SA et al. [69]	Patient similarity algorithms, ML for shared decision-making	Clinical decision aid tool developed for >4000 cancer survivors, integrating real-world clinical data	Shared decision-making and real-time ML-based clinical decision support	Clinical trial to evaluate improvement in cardio-oncology care using AI decision aid
	2023	Gao T et al. [70]	Interpretable ML for exercise prescription	Exercise prescription model based on ML and real-time monitoring in cardio-oncology	AI-driven exercise prescription system for preventive cardio-oncology care	Expected improvement in cardiovascular outcomes with AI-based exercise prescription
	2023	Wu S et al. [72]	Multimodal AI integrating wearables and voice assistants	Wearables + AI-driven risk detection of cardiotoxicity in multimodal clinical decision systems	AI-integrated multimodal symptom monitoring and risk detection	Clinician-validated AI system for continuous monitoring and early symptom detection
	2023	Papadopoulou SL et al. [73]	AI-assisted echocardiographic evaluation by oncology staff	AI-enabled handheld ultrasound device tested in 115 chemotherapy patients	AI-supported evaluation of cardiac function by oncology staff in chemotherapy patients	Automated LVEF calculation by oncology staff achieved sensitivity of 94–95%, specificity of 87–94%

**Table 3:** Scientific articles on AI in acute and subacute cardiovascular toxicity diagnosis and treatment optimization.

Category	Year of Study	Author	ML Method	Data Source	Application	Performance Metrics
Automated differential diagnosis	2024	Oikonomou EK et al. [57]	AI-enhanced ECG imaging for cardiac dysfunction risk stratification	Multicenter dataset of 1550 patients undergoing anthracycline or trastuzumab therapy	AI-enhanced ECG-based risk stratification of cancer therapy-related cardiac dysfunction	AI-ECG identified high-risk patients with 3.4× to 13.5× increased risk for cardiac dysfunction
	2024	Papadopoulou SL et al. [73]	AI-assisted evaluation of cardiac function using handheld ultrasound devices	115 chemotherapy patients evaluated for AI-enabled echocardiographic assessment	Automated LVEF assessment by oncology staff	Sensitivity: 94–95%, Specificity: 87–94% for LVEF detection using AI-enabled HUD
Treatment optimization by algorithms based on clinical data	2024	Zhou S et al. [76]	DL applied to longitudinal EHR data for heart disease risk prediction	Longitudinal electronic health records from breast cancer patients	Prediction of heart disease risk in breast cancer patients using DL models	AUC of 0.88 for predicting cardiovascular disease risk in breast cancer patients
	2022	Kappel C et al. [29]	AI-driven telemedicine platforms for equity in cardio-oncology care	Telemedicine applications for cardio-oncology care in rural communities	Improving access and equity in cardio-oncology using AI-driven telehealth solutions	Reported increase in access to cardio-oncology care in remote communities

**Table 4:** Scientific articles on AI in cardiovascular toxicity long-term detection and equity.

Category	Year of Study	Author	ML Method	Data Source	Application	Performance Metrics
Treatment optimization by algorithms based on clinical data	2024	Nechita LC et al. [33]	ML-enhanced ECG for long-term cardiotoxicity detection	ECG-based screening for subclinical cardiotoxicity in cancer survivors	Enhancing ECG interpretation for long-term detection of cardiotoxicity	Higher detection sensitivity of ECG-based AI screening for late-onset cardiotoxicity
	2023	Echefu G et al. [32]	AI applications in cardio-oncology for racial disparity analysis	Review of AI-driven healthcare disparities in cardio-oncology	Assessing AI’s role in reducing racial disparities in cardio-oncology	Recommendations for AI model bias mitigation and equitable risk assessment
	2023	Suero-Abreu GA et al. [80]	AI publication trends in cardiology and oncology research	Bibliometric analysis of AI trends in cardiology and oncology	Identifying research gaps and AI integration trends in cardiology-oncology	Growth analysis of AI research in cardio-oncology over the past decade

**Table 5:** Comparative analysis of key studies on AI and implantable devices in cardio-oncology.

Study	Key Findings	Device Type	AI Integration	Clinical Impact
Kappel et al. (2023) [29]	Explored AI-enhanced telemedicine for remote cardiac monitoring.	Various implantable devices	AI-enabled remote monitoring	Improved access to cardio-oncology care in rural areas.
Gao et al. (2022) [70]	Developed AI-driven models for CRT optimization in cancer survivors.	CRT	AI-assisted pacing adjustments	Improved treatment response and reduced heart failure exacerbations.
Riesenhuber et al. (2021) [85]	Examined pacemaker outcomes in cancer patients. Found increased mortality in post-diagnosis implantations.	Pacemaker	No AI	Highlighted need for early cardiac intervention.
Roy et al. (2021) [92]	Investigated cancer incidence in ICD recipients. Found no conclusive causal link.	ICD	No AI	Suggested potential interactions between device materials and cancer development.
Lee SY et al. (2021) [89]	Assessed CRT efficacy in chemotherapy-induced cardiomyopathy.	CRT	No AI	Found CRT beneficial in restoring cardiac function in cancer patients.
Munshi et al. (2013) [93]	Reviewed radiotherapy risks for cancer patients with pacemakers.	Pacemaker	No AI	Emphasized safety measures needed for radiation exposure.

**Table 6:** Wearable smart cardiac devices in cardio-oncology.

Study	Clinical Application	Device Type	Key Benefits	AI Integration
Hughes et al. (2023) [99]	Hypertension and Blood Pressure Monitoring	Omron HeartGuide, Withings BPM Core	Home-based blood pressure tracking, AI-driven trend analysis	Predictive analytics for hypertension risk, automated alerts
EHRA Guide, 2023 [100]	AI Integration and Advanced Functionalities	AI-driven ECG Analysis, DL Arrhythmia Detection	Automated arrhythmia classification, early warning systems	Personalized treatment recommendations, predictive analytics
Faro et al. (2022) [101]	Arrhythmia Detection and Management	KardiaMobile (AliveCor), Apple Watch ECG	Continuous arrhythmia monitoring, early AF detection	AI-powered AF detection, ML-based ECG interpretation
Sadler et al. (2022) [102]	Remote Patient Monitoring and Telemedicine	Fitbit, Garmin Smartwatches, BioSticker	Real-time cardiac data transmission, reduced clinic visits	Data aggregation for clinical decision support, AI-driven alerts
Bayoumy et al., Nat Rev Cardiol. (2021) [24]	Cardiotoxicity Surveillance	Zio XT Patch, Hexoskin Smart Shirt	Prolonged cardiac monitoring, early HF detection	AI-enhanced risk stratification for heart failure prediction

## Data Availability

No new data were created or analyzed in this study.

## Abbreviations

AF	Atrial Fibrillation
AI	Artificial Intelligence
AI-CTRCD	AI-Based Cancer Therapy-Related Cardiac Dysfunction Model
AI-ECG	AI-Enhanced Electrocardiogram
AI-EF	AI-Based Ejection Fraction Model
ANN	Artificial Neural Network
AUROC	Area Under Receiver Operating Characteristic Curve
BLE	Bluetooth Low Energy
CAD	Coronary Artery Disease
CardioMEMS	Wireless Hemodynamic Monitoring System
CNN	Convolutional Neural Network
CRT	Cardiac Resynchronization Therapy
CTRCD	Cancer Therapy-Related Cardiac Dysfunction
DL	Deep Learning
ECG	Electrocardiogram
EEG	Electroencephalography
EHR	Electronic Health Record
EHRA	European Heart Rhythm Association
EMG	Electromyography
GSR	Galvanic Skin Response
GANs	Generative Adversarial Networks
HF	Heart Failure
HUD	Handheld Ultrasound Device
ICD	Implantable Cardioverter Defibrillator
ICEDs	Implantable Cardiac Electronic Devices
IMU	Inertial Measurement Unit
KNN	K-Nearest Neighbors
LD	Linear Dichroism
LPWAN	Low Power Wide Area Network
LSTM	Long Short-Term Memory
LVEF	Left Ventricular Ejection Fraction
MCU	Microcontroller Unit
MI	Myocardial Infarction
ML	Machine Learning
MLP	Multilayer Perceptron
NLP	Natural Language Processing
PA	Pulmonary Artery
PCA	Principal Component Analysis
PET	Positron Emission Tomography
PPG	Photoplethysmography
RF	Random Forest
RNN	Recurrent Neural Network
SVM	Support Vector Machine
SoC	System-on-Chip
VEGF	Vascular Endothelial Growth Factor
XGBoost	Extreme Gradient Boosting
